# Ultrasound-Guided Sciatic Nerve Hydrodissection Can Improve the Clinical Outcomes of Patients with Deep Gluteal Syndrome: A Case-Series Study

**DOI:** 10.3390/diagnostics14070757

**Published:** 2024-04-02

**Authors:** Yun-Shan Yen, Chang-Hao Lin, Chen-Hao Chiang, Cheng-Yi Wu

**Affiliations:** 1 Department of Rehabilitation, Ditmanson Medical Foundation Chia-Yi Christian Hospital, Chiayi 600, Taiwan; longeryys@gmail.com; 2 Department of Orthopaedics, Ditmanson Medical Foundation Chia-Yi Christian Hospital, Chiayi 600, Taiwan; cych12763@gmail.com (C.-H.L.); chiangabaca@gmail.com (C.-H.C.)

**Keywords:** deep gluteal syndrome, sciatic nerve, nerve hydrodissection, ultrasound

## Abstract

Deep gluteal syndrome (DGS) is caused by sciatic nerve entrapment. Because fascial entrapment neuropathies may occur in multiple locations, ultrasound-guided nerve hydrodissection is a key component of DGS treatment. In this study, we examined the clinical outcomes of patients with DGS undergoing ultrasound-guided sciatic nerve hydrodissection. A 10 mL mixture consisting of 5% dextrose, 0.2% lidocaine (Xylocaine), and 4 mg betamethasone (Rinderon) was used for nerve hydrodissection. Clinical outcomes were evaluated using Numeric Rating Scale (NRS) scores of pain, the proportion of patients with favorable outcomes (reduction of ≥50% in pain), the duration for which patients exhibited favorable outcomes (percentage of follow-up duration), and the occurrence of major complications and minor side effects. A total of 53 patients were consecutively included and followed up for 3 to 19 months. After the initial injection, the NRS scores significantly improved at 1 week, 1 month, 3 months, and the final follow-up. Specifically, 73.6%, 71.7%, 64.2%, and 62.3% of the patients exhibited favorable outcomes at 1 week, 1 month, 3 months, and the final follow-up, respectively. The median duration for which the patients exhibited favorable outcomes was 84.7% of the follow-up period. Three patients (5.7%) experienced transient dizziness and vomiting, which resolved without further treatment. No vessel or nerve puncture was observed. Overall, ultrasound-guided sciatic nerve hydrodissection is a safe procedure that mitigates the pain associated with DGS. To achieve favorable outcomes, three consecutive injections 3 weeks apart are required.

## 1. Introduction

Low back pain and sciatica are common causes of disability [1]. Although much research attention has been given to nerve root compression by disc herniation and stenosis in the lumbar spine, up to 49% of sciatica cases exhibit no lesions involving impinging nerve roots on spinal magnetic resonance images [2]. However, musculoskeletal disorders in the deep gluteal space can lead to compression of the sciatic nerve, contributing to pain in the glutes and posterior hip. Entrapment of the sciatic nerve in the deep gluteal space is known as deep gluteal syndrome (DGS).

The causes of sciatic nerve entrapment can be classified as follows: (1) causes related to fibrous and fibrovascular bands, including compressive or bridge-type bands, adhesive bands or horse-strap bands, and bands anchored to the sciatic nerve with undefined distribution; (2) piriformis muscle pathology, such as hypertrophy of the piriformis muscle, dynamic entrapment by the piriformis muscle, anatomical variations between the piriformis muscle and sciatic nerve, anomalous attachments in proximal or distal insertions, and trauma or overuse; (3) gemelli-obturator internus syndrome; (4) causes related to the quadratus femoris and ischiofemoral pathology; (5) hamstring pathology; (6) gluteal pathology; and (7) changes in the alignment of the pelvis, femur, and spine, which may affect normal excursion of the sciatic nerve [3].

Before the sciatic nerve is located, the anatomy of the deep gluteal space must be understood. When observed from the posterior, it is delimited (in order from superficial to deep) by the gluteus maximus, posterior ilium, superior ramus and body of the ischium, posterior acetabulum, and anterior aspect of the femoral head and femoral neck. The lateral boundaries include the intertrochanteric crest and the greater trochanter, and the medial boundaries include the posterior superior iliac spine, sacroiliac joint, greater and lesser sciatic notches, sacrotuberous ligament, and ischial tuberosity. Superiorly, the sciatic nerve is delimited by the iliac crest. The ischiofemoral tunnel, which is located between the deep gluteal space and the posterior thigh compartment, serves as the lower boundary of the deep gluteal space. When the deep gluteal space is viewed from superior to inferior, the muscles (in order) are the gluteus maximus, gluteus medius, gluteus minimus, piriformis, superior gemellus, obturator internus, inferior gemellus, and quadratus femoris. The sciatic nerve enters the deep gluteal space through the greater sciatic foramen, and it courses anterior to the piriformis muscle and then inferiorly and posteriorly to the superior gemellus, obturator internus, inferior gemellus, and quadratus femoris. Finally, the sciatic nerve enters the ischiofemoral tunnel between the ischial tuberosity and greater trochanter [3,4,5,6].

Generally, conservative treatment is initially used to manage DGS. Such treatment includes rest, activity modification, administration of muscle relaxants, administration of oral anti-inflammatory medicines, and physical therapy [3,5,6]. However, when such treatment is ineffective, local anesthesia at the pain site and nerve hydrodissection are implemented before surgery. The effectiveness of local anesthesia for pain reduction has been confirmed in several randomized controlled trials of thiocolchicoside, triamcinolone, botulinum toxin A, lidocaine, and clonidine [7,8,9,10]. However, because fascial entrapment neuropathies may occur in multiple locations, blocking at a specific site may provide partial pain relief for patients [11]. Nerve hydrodissection is a technique that involves injecting a large volume of solution into areas of suspected compression to release the compressed nerve from the surrounding fascia and soft tissues [12]. This technique is both effective and safe for patients with ulnar neuropathy at the elbow and carpal tunnel syndrome [13,14,15,16,17,18,19,20,21,22]. Despite the benefits of nerve hydrodissection, few studies have examined the clinical outcomes of ultrasound-guided sciatic nerve hydrodissection, with no studies reporting on changes in pain levels during the follow-up period [23,24,25]. Therefore, in this study, we examined the clinical outcomes of ultrasound-guided sciatic nerve hydrodissection over a follow-up period of at least 3 months.

## 2. Materials and Methods

### 2.1. Patients

Ethical approval for this study was obtained from the Ethical Review Board of Ditmanson Medical Foundation Chia-Yi Christian Hospital (approval no. 2023086). In this retrospective case-series study, patients who underwent DGS at a single hospital during the period from August 2017 to February 2023 were consecutively included. All patients experienced pain for at least 3 months, with limited pain relief after 1 month of physical therapy and analgesic medication. DGS was diagnosed in accordance with the following criteria: (1) nondiscogenic pain in the posterior hip that becomes more intense when the patient remains seated for over 30 min; (2) positive findings from a seated piriformis stretch test; and (3) abnormal findings in the deep gluteal space after pelvic radiography, pelvic magnetic resonance imaging, and spinal magnetic resonance imaging. Patients who met any of the following criteria were excluded: receiving previous injections in the lumbar spine or hip within 6 months; having a history of lumbar spinal stenosis; having lumbar disc herniation, avascular hip necrosis, or hip osteoarthritis; and being followed up for less than 3 months.

### 2.2. Procedures

The injection method is briefly described as follows. All procedures were performed by an experienced physician in ultrasound-guided settings, and all treatments were delivered in an outpatient setting. Ultrasound-guided nerve hydrodissection was performed using a LOGIQ S8 ultrasound system (GE Healthcare, Milwaukee, WI, USA) equipped with a 3–12-MHz linear probe (1–6-MHz curve probe).

After the patient assumed a prone position with their lower extremities in neutral rotation, their posterior gluteal area was disinfected with povidone-iodine (Betadine). Their skin was subsequently allowed to dry for 30 s while aseptic drapes were prepared for the procedure. The ultrasound probe was wrapped with a sterile polyurethane film, and its inner surface was filled with ultrasound transmission gel. A second round of disinfection at the injection site and the contact medium for ultrasound-guided hydrodissection was performed using a solution containing 2% chlorhexidine gluconate (*w*/*v*) in 75% alcohol (*v*/*v*).

To locate the sciatic nerve, the probe was initially placed to identify the ilium, which is a laterally descending hyperechoic oblique bony structure. Once the ilium was visualized, the ultrasound probe was moved downward from the ilium, gradually along the outer edge of the pelvis. The gluteus maximus muscle lies posteriorly to the ilium. Then, the probe was positioned beneath the gluteus maximus muscle to visualize the piriformis muscle, under which the sciatic nerve runs. Subsequently, the lower extremity was passively internally and externally rotated with the knee in a flexed position. Adjusting the angle and position of the probe as necessary ensured optimal visualization of the nerve’s course from beneath the piriformis muscle to above the short rotator muscle group. This movement allowed for dynamic visualization of the sciatic nerve. The nerve was observed at the back of the thigh between the long head of the biceps femoris muscle laterally and the semimembranosus muscle medially on the adductor magnus, and it was visualized as a hyperechoic image. Subsequently, the nerve was proximally followed until it reached the anatomic plane between the gluteus maximus and pelvitrochanteric muscles. Optimal images of the nerve and the surrounding vessels near its border were obtained through probe manipulation and power-mode color Doppler ultrasonography.

**Figure 1:**
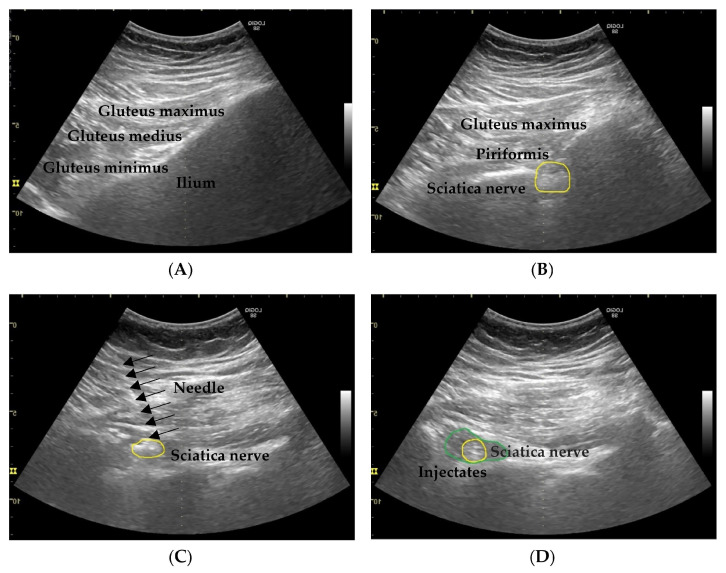
Ultrasound images of sciatic nerve hydrodissection. (**A**) The ultrasound probe was carefully positioned to identify the ilium, a laterally descending hyperechoic oblique bony structure. (**B**) When the ilium was traced, a notable gap was observed, representing the greater sciatic foramen and indicating the trajectory of the sciatic nerve. The nerve was visualized at a deep level, beneath the gluteus maximus and piriformis muscles, extending laterally toward the greater trochanter. In the image, the yellow circle indicates the sciatic nerve. (**C**) Because of the superior gemellus muscle, the sciatic nerve was separated from the ischium. A needle (the area indicated by the arrow) was carefully inserted from the lateral to the medial side, targeting the sciatic nerve. (**D**) After the needle’s tip was confirmed to be close to the designated area, a 10 mL solution containing 5% dextrose in water (D5W), 0.2% lidocaine (Xylocaine), and 4 mg of betamethasone (Rinderon) was injected under real-time ultrasound guidance to hydrodissect the sciatic nerve. In the image, the green circle indicates the area to which the solution spread.

After precise localization of the sciatic nerve, hydrodissection targeting the nerve was performed. A 50 mm 23G needle was guided under ultrasound visualization to the vicinity of the sciatic nerve and inserted at a 45° angle into the skin in the longitudinal plane. After the needle was inclined at an angle of 5° toward the lateral side to achieve a slight lateral-to-medial approach, it was advanced until it reached the region immediately lateral to the sciatic nerve within the musculoaponeurotic plane between the gluteus maximus and pelvitrochanteric muscles. While paying attention to the surrounding vessels, the physician placed the tip of the needle on the medial side of the nerve to hydrodissect the soft tissue around the nerve to the point where the injectate surrounded the entire sciatic nerve. After performing careful aspiration and confirming the absence of abnormal findings, the physician injected a 10 mL mixture containing 5% dextrose in water (D5W), 0.2% lidocaine (Xylocaine), and 4 mg of betamethasone (Rinderon) into the target area under real-time ultrasound guidance to hydrodissect the sciatic nerve and effectively mitigate the patient’s pain with fluid surrounding the nerve. Figure 1 shows ultrasound images of the sciatic nerve hydrodissection.

After the procedure, the patient was monitored for 10 min for any clinical manifestations, including pain at the injection site, experiencing a metallic taste, dizziness, tachycardia, full-body paresthesia, auditory changes, slurred speech, or motor ataxia. All patients were scheduled to receive three consecutive injections 3 weeks apart. No additional physical therapies or analgesic medications were administered following the initial injection.

### 2.3. Outcome Evaluation

The primary outcomes were the reduction in pain in the posterior hip region and the persistence of favorable outcomes. Pain reduction was evaluated using Numeric Rating Scale (NRS) scores ranging from 0 to 10. The percentages of patients who exhibited favorable outcomes (a reduction of ≥50% in pain) were calculated at 4 time points: 1 week, 1 month, 3 months, and the final follow-up. To evaluate the persistence of favorable outcomes, the duration for which each patient exhibited favorable outcomes was recorded. However, the counting of days undergoing favorable outcomes might be limited because the follow-up periods of all of the patients were not equal. Therefore, we calculated the persistence of favorable outcomes by dividing the number of days during which the patients exhibited favorable outcomes by the total number of follow-up days ($\frac{Days\;of\;exhibition\;of\;a\;favorable\;outcome\;(days)}{Days\;of\;follow\text{-}up\;(days)}$). This calculation provided insights into the duration for which the patients exhibited favorable outcomes throughout the follow-up period. The secondary outcomes were major complications (vessel or nerve puncture) and minor side effects (dizziness, vomiting, skin bruises, local tenderness, itchiness, and allergic reactions).

### 2.4. Statistical Analysis

Repeated measures of NRS for pain were compared at different time points with a linear generalized mixed model. A subgroup analysis was conducted to compare patients who achieved favorable outcomes at the final follow-up versus those who did not. Differences in continuous and discrete variables between the 2 groups were determined using *t* and χ^2^ tests. All statistical analyses were conducted using IBM SPSS Statistics version 28.0.1.0 (IBM, Armonk, NY, USA).

## 3. Results

**Figure 2:**
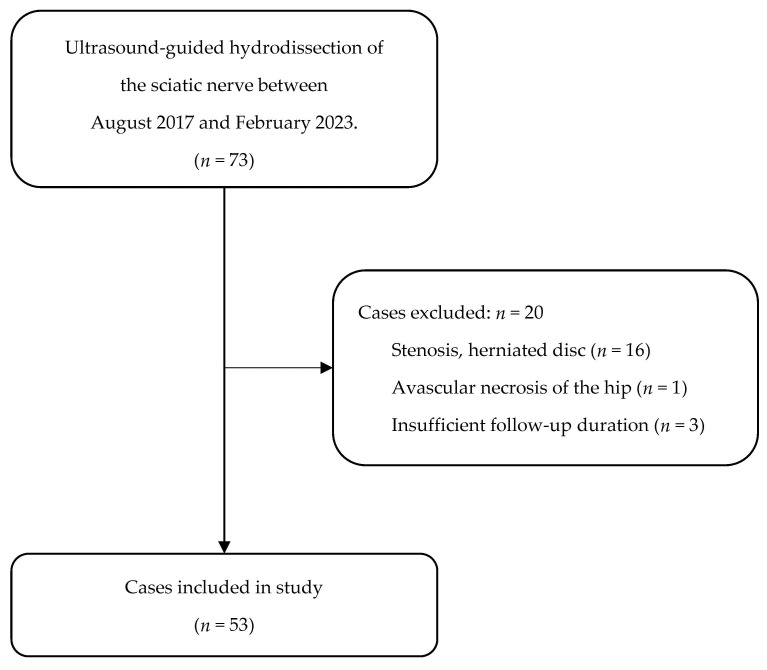
Flowchart of patient selection.

Of a total of 73 patients, 20 were excluded: 16 patients had a history of lumbar spinal stenosis and lumbar disc herniation, 1 patient had a history of avascular hip necrosis, and 3 patients were lost to follow-up (Figure 2). Ultimately, 53 patients were included in the study, for a mean follow-up duration of 5.4 ± 4.0 months (range: 3 to 19 months). Of these patients, 19 (35.8%) were men, with a mean age of 58.2 ± 15.1 years (range: 24 to 84 years) (Table 1).

The NRS score of pain before the procedure was 6.4 ± 1.6. This score significantly increased to 3.1 ± 1.8, 3.0 ± 1.8, 3.1 ± 2.0, and 3.0 ± 2.1 at 1 week, 1 month, 3 months, and the final follow-up, respectively. During the follow-up period, the percentage of patients exhibiting favorable outcomes gradually decreased over time. Specifically, 92.5% of the patients exhibited favorable outcomes immediately after the initial injection, but this percentage decreased over subsequent assessments. At the final follow-up, only 62.3% of the patients were found to retain their favorable outcomes (Table 2). The mean persistence of favorable outcomes was calculated to be 68.4% ± 38.4% (median: 84.7%, interquartile range: 60.3%). No major complications were observed. Three patients experienced transient dizziness, which resolved without further treatment within 4 h.

Table 3 presents the risk factor analysis results of unfavorable outcomes at the final follow-up. In the unfavorable group, 90% of the patients received one or two injections. This percentage was significantly higher than that in the favorable group.

## 4. Discussion

In this study, we observed that patients who underwent ultrasound-guided sciatic nerve hydrodissection for DGS experienced a significant reduction in pain, and this reduction persisted throughout the majority of the follow-up duration. None of the patients experienced major complications, and only three patients experienced minor side effects, indicating the safety of our procedure and injectate combination.

According to our literature review, only two studies have reported a notable reduction in pain after ultrasound-guided sciatic nerve hydrodissection for DGS. In these studies, the mean NRS scores of pain were 4.7 and 8.3 before the procedure and decreased to 0.5 and 2.8, respectively, after the procedure [23,24]. In our study, 49 patients (92.5%) immediately experienced a reduction of ≥50% in pain after the procedure, with overall mean NRS scores of 6.4, 3.1, 3.0, 3.1, and 2.7 before treatment, at 1 week, at 1 month, at 3 months, and at the final follow-up, respectively. Overall, our results after the procedure were consistent with those of previous studies. Among our patients, 15 were followed up for over 6 months. Of these patients, 13 (86.7%) were found to retain their favorable outcomes at the final follow-up. Further research involving additional cases is required to thoroughly confirm these findings.

We compared the outcomes between nerve hydrodissection and local injection for DGS. Three randomized control trials have reported local injections to be effective for treating DGS. The mean NRS scores at pretreatment ranged from 6.7 to 7.4 and decreased to a range of 1.9 to 4.7 at 1 month after treatment and 0.9 to 4 at 3 months after treatment [7,8,9]. The variation in the treatment effects might be attributable to the different injectates used in the trials, which included thiocolchicoside, triamcinolone, botulinum toxin A, lidocaine, betamethasone, clonidine, and bupivacaine. Our results regarding pain reduction were comparable to those observed in patients treated with local injections.

Several injectate combinations have been used for sciatic nerve hydrodissection. Burke et al. [23] reported an approach involving the injection of 1 to 2 mL of saline, followed by the injection of a mixture of 1 mL of 1% lidocaine with either 1 mL of Kenalog-40 or 1.5 mL of Celestone Soluspan (9 mg), followed by the injection of 5 to 10 mL of sterile saline. Rosales et al. [24] reported an approach involving the injection of a mixture of 1 mL of corticosteroid solution (40 mg of methylprednisolone) diluted in 4 mL of 2% lidocaine and 20 mL of saline. In this study, we used a 10 mL mixture of 5% in dextrose, 0.2% lidocaine (Xylocaine), and 4 mg of betamethasone (Rinderon) as an injectate. Dextrose in water (D5W) is a commonly used injectate in hydrodissection, with an analgesic effect on the treatment of peripheral entrapment neuropathies [26]. Low-dose lidocaine is typically used to avoid patient discomfort during injection [27]. Betamethasone (Rinderon) has a favorable pain reduction effect and is effective and safe for use in selective nerve root blocking [28,29]. In a previous study, Moshrif and Elwan [30] used a mixture of D5W and steroids for the treatment of plantar fasciitis and reported that D5W resulted in a significant reduction in pain during the procedure. In our study, three patients experienced minor side effects (mainly dizziness) after the procedure, presumably because we used betamethasone and lidocaine. Given that both betamethasone and lidocaine were used at low doses, these minor side effects resolved without further treatment. Overall, our results indicated that our injectate combination resulted in an adequate reduction in pain with a low incidence of minor side effects, indicating its efficacy and safety.

In addition to minor side effects from the injectate, several major complications may occur, including infection, vessel puncture, and nerve puncture. However, the occurrence of these complications is rare. In previous studies on ultrasound-guided hydrodissection for DGS that involved a total of 91 patients, no local or general complications were reported [23,24,25]. In the present study of 53 patients, these major complications were also not observed. Therefore, performing ultrasound-guided hydrodissection for DGS is safe. The risks associated with major complications can be minimized by employing care during procedures and taking precautions. For infection prevention, skin must be properly disinfected before injection and appropriate aseptic techniques must be used during injection. In addition, understanding the anatomical structures at the injection site can minimize the risks of vessel and nerve puncture, and using power-mode color Doppler ultrasonography can prevent vessel puncture. Pain and swelling at the injection site may lead to decreased patient satisfaction. However, previous studies have reported rare incidences, and these issues could resolve spontaneously within a short period [22,31].

To investigate the potential risk factors associated with unfavorable outcomes, we compared patients who achieved favorable outcomes to those who achieved unfavorable outcomes at the final follow-up. We discovered that a repeated injection after the initial injection was the most significant factor associated with pain reduction. These findings are consistent with those of a previous study, which reported that a repeated injection 2 to 3 weeks after the initial injection contributed to a prolonged clinical reduction in pain [32]. Therefore, repeated injections are recommended for patients with DGS to maximize their clinical benefits. According to our results, receiving over three consecutive injections 3 weeks apart may be an optimal approach for achieving favorable outcomes at the final follow-up. In locations where nerves were compressed, although the initial injection expanded the space around the nerves, this condition was unstable, and compression recurred later. After three rounds of injection, compression became unlikely to recur.

This study has some strengths. First, we used more parameters than those used in previous studies to represent pain reduction, and our minimum follow-up period was 3 months. Second, we calculated the persistence of favorable outcomes to determine the duration of treatment effects. This calculation enabled us to determine the duration for which our patients exhibited a reduction of ≥50% in pain.

This study has some limitations. For instance, because we did not designate a control group to receive conservative treatment (D5W or steroids alone), we were unable to evaluate the effectiveness and synergistic effects of different treatments. In addition, we were unable to comprehensively evaluate the activity levels after the treatment of our patients through quantitative parameters, particularly in cases with extended follow-up periods. These activity levels may have affected the clinical outcomes of the procedure, thereby serving as a confounding factor. Given these limitations, although our findings are applicable to patients with DGS, these findings should be interpreted with caution. Further studies with comparative designs, larger sample sizes, and longer follow-up periods are warranted to validate our findings.

## 5. Conclusions

Ultrasound-guided sciatic nerve hydrodissection is a safe procedure that mitigates the pain associated with DGS. Injecting a 10 mL mixture of 5% dextrose, 0.2% lidocaine (Xylocaine), and 4 mg of betamethasone (Rinderon) is considered appropriate for nerve hydrodissection. To achieve favorable outcomes, at least three consecutive injections 3 weeks apart should be performed.

## Figures and Tables

**Table 1:** Patient characteristics.

*n*. of Patients	53
Male	19 (35.8%)
Female	34 (64.2%)
Age (years)	
Mean ± sd	58.2 ± 15.1
Median (IQR)	59.0 (22.0)
*n*. of injections	
1	20 (37.7%)
2	15 (28.3%)
3 and above	18 (34.0%)
Length of follow-up (months)	
Mean ± sd	5.4 ± 4.0
Median (IQR)	3.0 (3.5)
Min–Max	3–19

IQR: interquartile range.

**Table 2:** Pain improvement during the follow-up periods.

Parameters	Values		*p*-Value
NRS of pain (0–10)			
Pretreatment	Mean ± sd:	6.4 ± 1.6	Ref.
	Median (IQR):	6 (2.5)	
1 week	Mean ± sd:	3.1 ± 1.8	<0.001 *
	Median (IQR):	2.5 (3)	
1 month	Mean ± sd:	3.0 ± 1.8	<0.001 *
	Median (IQR):	2.5 (2.5)	
3 months	Mean ± sd:	3.1 ± 2.0	<0.001 *
	Median (IQR):	2.5 (3.25)	
Final follow-up	Mean ± sd:	3.0 ± 2.1	<0.001 *
	Median (IQR):	2.5 (3.25)	
Pain reduction ≥ 50% (*n*, %)			
Post-procedure	49 (92.5%)		
1 week	39 (73.6%)		
1 month	38 (71.7%)		
3 months	34 (64.2%)		
Final follow-up	33 (62.3%)		

* The follow-up values were compared with the pretreatment values. IQR: interquartile range.

**Table 3:** Risk factor analysis results of unfavorable outcomes at the final follow-up.

	Unfavorable	Favorable	*p*-Value
	20	33	
Age			
Mean ± sd	58.6 ± 17.4	58.0 ± 13.8	0.899
Median (IQR)	58.5 (26.5)	60 (18.5)	
Pain at pretreatment (0–10)			
Mean ± sd	6.3 ± 1.5	6.5 ± 1.6	0.651
Median (IQR)	5.25 (2.5)	6 (2.5)	
*n*. of injections during the follow-up period			
1	11 (55.0%)	8 (24.2%)	0.008
2	7 (35.0%)	8 (24.2%)	
3 and above	2 (10.0%)	17 (51.6%)	

IQR: interquartile range.

## Data Availability

The data that support the findings of this study are available from the corresponding author, C.-Y.W., upon reasonable request.
